# ‘BRS Vitoria’ Grapes Across Four Production Cycles: Morphological, Mineral, and Phenolic Changes

**DOI:** 10.3390/plants14060949

**Published:** 2025-03-18

**Authors:** Mariana de Souza Leite Garcia-Santos, Victoria Diniz Shimizu-Marin, Yara Paula Nishiyama-Hortense, Carolina Olivati, Reginaldo Teodoro de Souza, Francielli Brondani da Silva, Natália Soares Janzantti, Ellen Silva Lago-Vanzela

**Affiliations:** 1 Institute of Biosciences, Humanities and Exact Sciences, São Paulo State University, Cristóvão Colombo 2265, São José do Rio Preto 15054-000, SP, Brazil; mariana.g.santos@unesp.br (M.d.S.L.G.-S.); victoria.shimizu@unesp.br (V.D.S.-M.); yaranishy@gmail.com (Y.P.N.-H.); carolinaolivati@gmail.com (C.O.); francielli.brondani@unesp.br (F.B.d.S.); natalia.soares-janzantti@unesp.br (N.S.J.); 2 Brazilian Agricultural Research Corporation Grape and Wine, Via Acesso Euphly s/n, Jales 15700-000, SP, Brazil; reginaldo.souza@embrapa.br

**Keywords:** Brazilian grape, anthocyanin, minerals, morphological characterization, rootstocks

## Abstract

The ‘BRS Vitoria’ grape has sensory characteristics that favor its consumption. However, different rootstocks and harvest periods can directly influence its phenolic composition, physicochemical and morphological characteristics, and mineral content. This study evaluates the mineral and anthocyanin composition of the ‘BRS Vitoria’ grape from a production cycle (PC1: ‘IAC 572’ rootstock, main harvest) and compares its physicochemical, morphological, and mineral characteristics to other cycles (PC2: ‘Paulsen 1103’ rootstock, second harvest; PC3: ‘IAC 572’ rootstock, second harvest; and PC4: ‘Paulsen 1103’ rootstock, main harvest), highlighting its potential for use and providing initial insights into the influence of rootstocks and environmental conditions. PC1 grapes contained important amounts of potassium, phosphorus, calcium, magnesium, iron, manganese, and zinc (345.16, 50.50, 20.34, 13.61, 0.54, 0.27, and 0.03 mg⋅100 g^−1^, respectively), and a complex anthocyanin profile, predominantly derived from malvidin, which supports their use in processing due to the thermal stability. In the second part of the study, PC2 grapes stood out for their skin percentage and acidity. PC3 grapes exhibited higher values in parameters associated with size, mass, and mineral content, which may have been influenced by the use of the ‘IAC 572’ rootstock. PC4 grapes showed the highest maturation index (38.68), total phenolic compounds (1750.88 mg EGA⋅kg^−1^), and total monomeric anthocyanins (742.86 mg mv-3,5-glc⋅kg^−1^). These results may have been influenced by the environmental conditions during the main harvest season. Bunches from all cycles were cylindrical, very compact, with dark red-violet berries and featuring thick skin with pruine and firm colorless, seedless flesh. The study of the influence of these factors is complex due to the impact of various other variables and the synergistic effect between them. Despite physicochemical and morphological differences, ‘BRS Vitoria’ grapes from different PCs are suitable for fresh consumption and processing, potentially as a nutraceutical ingredient.

## 1. Introduction

Grapes and non-alcoholic grape-derived products are widely consumed worldwide and are increasingly recognized for their potential as nutraceutical foods [1,2]. This trend is attributed to the presence of macro- and micronutrients, including vitamins and minerals, as well as bioactive compounds, such as phenolic compounds (e.g., anthocyanins, flavonols, hydroxycinnamic acids derivatives, flavan-3-ols, and stilbenes) [2,3].

In Brazil, grape production has gained economic prominence due to increased domestic consumption and its significant role in the international market. Data from the Brazilian Institute of Geography and Statistics show that the value of grape production in the country reached 5.31 million BRL in 2023, approximately 1.2 times higher than in the previous year, with a total of around 1.76 million tons produced [4]. In the same year, the International Organisation of Vine and Wine (OIV) reported that Brazil ranked eighth both among the largest grape producers and the largest consumers of table grapes [5]. With the expansion of grapevine cultivation and the need to adapt to the diverse soil and climate conditions across Brazil, the Brazilian Agricultural Research Corporation (EMBRAPA, Brasilia, Brazil) has heavily invested in developing versatile cultivars. Among these, the seedless grape ‘BRS Vitoria’ (‘CNPUV 681-29’ [‘Arkansas 1976’ × ‘CNPUV 147-3’ (‘White Niagara’ × ‘Venus’)] × ‘BRS Linda’) stands out [6]. This cultivar, a non-*V. vinifera* interspecific hybrid, is highly valued in both national and international markets. Given the multifunctional role of grapes as a health promoter, this cultivar can be better explored as a source of phenolic compounds and minerals [1].

The ‘BRS Vitoria’ grape is cultivated in various grape-growing regions across Brazil due to its growing consumer demand, excellent climatic adaptation, high mildew tolerance, early production cycle (PC), high bud fertility, and remarkable productivity. This cultivar is available year-round in the market [3,7,8]. However, the characteristics of the grape can be influenced by the choice of rootstock. Using grafted rootstocks is a common practice in grapevine cultivation in Brazil, as they can adapt to adverse soil conditions [9]. Among the most widely used in the country are ‘IAC 572’ ((*Vitis riparia* × *Vitis rupestris*) × *Vitis caribaea*) and ‘Paulsen 1103’ (*Vitis berlandieri* × *Vitis rupestris*). ‘IAC 572’ exhibits high vigor, excellent root development, and adaptability to both sandy and clayey soils. In contrast, the ‘Paulsen 1103’ rootstock stands out for its resistance to fusariosis [10,11]. It is characterized by its ability to increase vine vigor and delay maturation, its tolerance to dry and saline soils, and its preference for clayey soils. Properly selecting rootstock plays a crucial role in the success of grapevine cultivation, as it requires compatibility and affinity between the rootstock and the scion cultivar [11].

The choice of rootstock can influence the vigor of ‘BRS Vitoria’, which is moderate, as well as the productivity and bunches and berries’ physicochemical and morphological characteristics. For example, excessive vegetative vigor can create a microclimate with high humidity and low radiation, increasing the bunches’ shading and hindering the accumulation of soluble solids in the berries [10]. Additionally, rootstocks can directly influence the scion’s photosynthetic activity [12] and vary in their capacity to absorb and translocate water and nutrients [13], affecting the chemical composition of the grapes. Calili et al. [10] also found that the Paulsen 1103 rootstock promotes higher accumulation of soluble solids and a higher maturation index, while the ‘IAC 572’ and ‘IAC 766’ rootstocks accelerate vine precocity compared to ‘Paulsen 1103’ during early phenological phases.

Besides the cultivar and rootstock influence, fruit characteristics are also the result of various external factors, such as management practices and edaphoclimatic conditions [3]. Several studies evaluating the influence of rootstocks highlight that the effect of seasonal climatic fluctuations is often considered to have a greater impact on the agronomic traits of the vine than the rootstock itself [10,14]. For ‘BRS Vitoria’ grapes grown in the state of São Paulo, Brazil, over two consecutive years, Callili et al. [10] observed a lower accumulation of soluble solids in the grapes during the year with higher precipitation during berry maturation, especially near the harvest date, for three different rootstocks tested. Anastasiou et al. [15] also reported a strong correlation between climatic parameters and the yield and quality properties of table grapes from Greece. They also observed that the effect of soil conditions varied depending on the climatic conditions.

Therefore, evaluating the fruit quality under different production cycles and rootstocks is crucial to understanding their combined impact on ‘BRS Vitoria’ grape composition and quality parameters. Building on this context, this study aimed to characterize different PCs of the ‘BRS Vitoria’ cultivar grown in the northwestern region of São Paulo. In the first PC (PC1), the qualitative and quantitative profiles of anthocyanins and mineral composition were analyzed using, respectively, high-performance liquid chromatography with a diode array detection coupled to electrospray ionization mass spectrometry (HPLC–DAD–ESI-MS/MS) and inductively coupled plasma mass spectrometry (ICP-MS). Subsequently, three additional PCs of this cultivar were evaluated to assess the variability in certain physicochemical and morphological characteristics, which are scarcely documented in the literature for ‘BRS Vitoria’. These findings contribute to this cultivar’s valorization and provide a foundation for developing nutraceutical ingredients and health-oriented food products.

## 2. Results and Discussion

### 2.1. Qualitative and Quantitative Profile of Anthocyanins and Mineral Composition of ‘BRS Vitoria’ Grapes from PC1

The ‘BRS Vitoria’ grape had a moisture content (75.71 ± 0.17) under the range described in the literature (77–87%) for other cultivars [3,16]. One of the leading quality indicators for table grapes is the soluble solids (SS), which is directly related to ripening and taste/flavor. It can be noted that PC1 grapes had an SS content (22.10 ± 0.10 °Brix) higher than that recommended (19.0 ºBrix) by Maia et al. [8] and reported by Colombo et al. [7] (16.53 to 17.57 °Brix) and Martinelli et al. [17] (17.41 ºBrix) for the same cultivar. The ‘BRS Vitoria’ grape also showed a total acidity (TA) of 0.68 ± 0.00 g tartaric acid⋅100 g^−1^ and pH of 3.24. Nishiyama-Hortense et al. [18] reported values similar to those found herein for ‘BRS Vitoria’ grapes from Marialva, state of Paraná, southern Brazil (TA of 0.72 g tartaric acid⋅100 g^−1^ and pH 3.62). Martinelli et al. [17] reported a TA of 0.48 g tartaric acid⋅100 g^−1^ and pH of 4.05 for that same cultivar from the semiarid region of Minas Gerais State, Brazil. The grape had an SS/TA ratio of 32.5 ± 0.24 with these results. Considering the international market, which requires a maturation index of at least 20, the ‘BRS Vitoria’ grapes from PC1 were suitable for fresh market sales.

From this PC1, which showed an adequate maturation stage, a detailed evaluation of the composition of minerals and anthocyanins was carried out. The ‘BRS Vitoria’ grapes showed interesting amounts of potassium (K), phosphorus (P), calcium (Ca), magnesium (Mg), iron (Fe), manganese (Mn), and zinc (Zn) (Table 1). The K content was higher than that reported for Meili (270–300 mg K⋅100 g^−1^) [19] and Chardonnay grapes (247 mg K⋅100 g^−1^) [20]. P was the second most abundant macromineral found in PC1, followed by Ca and Mg. The P content was higher than that reported for the Chardonnay grape (19.19 mg Mg⋅100 g^−1^) [20]. The Ca and Mg concentrations were similar to those found for the Meili grape (14–24 mg Ca⋅100 g^−1^ and 11–18 mg Mg⋅100 g^−1^, respectively) [19] and higher than those reported for the Cabernet Sauvignon and Merlot grapes, which had 3.29 and 4.94 mg Ca⋅100 g^−1^, and 3.89 and 5.08 mg Mg⋅100 g^−1^, respectively [21].

The most abundant micromineral in the ‘BRS Vitoria’ grape was Fe, followed by Mn. The lowest concentrations were observed for Zn. The concentrations of Fe were higher than those reported in the literature for the grapes Cabernet Sauvignon (0.08 mg Fe⋅100 g^−1^) and Merlot (0.20 mg Fe⋅100 g^−1^) by Panceri et al. [21]. Given the results, it can be highlighted that the ‘BRS Vitoria’ grape has a higher concentration of minerals than many traditionally marketed grapes. This cultivar is recommended as an ingredient in different products to contribute significantly to the recommended dietary allowances (RDA) of minerals.

Regarding the analyses related to the qualitative profile of anthocyanins from ‘BRS Vitoria’ grapes, 33 compounds were identified (Table 2). A complex profile of anthocyanins is noted, similar to that of Colombo et al. [3], for the same cultivar, including identifying some anthocyanins in *cis* and *trans* conformations. Six main anthocyanidins (aglycones) commonly present in grapes were detected—delphinidin (dp), cyanidin (cy), petunidin (pt), peonidin (pn), malvidin (mv), and pelargonidin (pl)—by their molecular mass (*m*/*z* 303, 287, 317, 301, 331, and 271, respectively) obtained from ionization products in mass spectrometry (MS/MS). This cultivar is a hybrid (between *V. vinifera* and *V. labrusca*) and contains monoglycosylated and diglycosylated anthocyanins, with close molar percentages (45.5 and 54.5%, Table 2).

The six complete series of aglycones (dp, cy, pt, pn, mv, and pl) detected in ‘BRS Vitoria’ were identified for their 3-glucoside-*p*-coumaroyl (3-cmglc). Furthermore, the series of five aglycones (dp, cy, pt, pn, and mv) were detected for their non-acylated 3,5-diglucoside (3,5-diglc), 3,5-diglucoside-coumaryl (3-cmglc-5-glc), as well as the diglycosylated acetylated (3-acglc-5-glc) derivative derived from mv; the monoglucosylated anthocyanins were also identified for their non-acylated (3-glc) and 3-glucoside-acetyl (3-acglc). Notably, most anthocyanins are derived from mv, totaling approximately 50%, emphasizing mv-3,5-diglc (~16%) and mv-3-*trans*-cmglc-5-glc (~17%).

Colombo et al. [7] carried out a study on the composition of anthocyanins in the ‘BRS Vitoria’ grape produced in Paraná, Brazil; the authors also reported the majority presence of mv, as well as of pn. These compounds derived from mv are trisubstituted methoxylated anthocyanins and are commonly more resistant to thermal degradation than other anthocyanins, such as dp (trisubstituted non-methoxylated). This grape also had an important proportion of acylated anthocyanins (55%), of which 51% were coumaroyl. The total anthocyanin concentration, expressed as mv-3,5-diglc equivalents, was 202.28 ± 22.31 mg⋅kg^−1^. The presence of anthocyanins with chemical structures less susceptible to thermal and oxidative degradation would encourage their use as raw materials for developing differentiated products and nutraceutical ingredients.

### 2.2. Physicochemical, Morphological, and Mineral Characterization of ‘BRS Vitoria’ Grapes Across Different PCs

**Figure 1:**
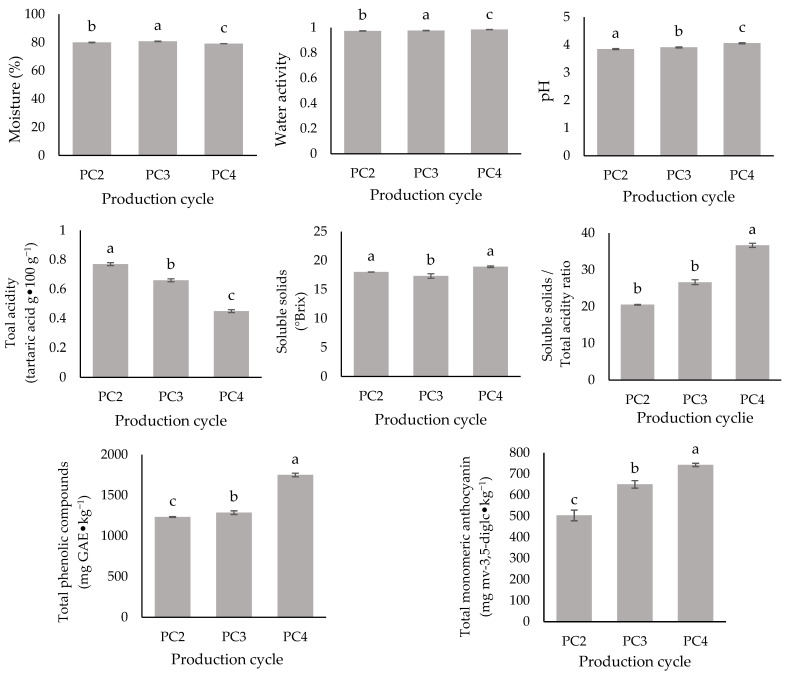
Basic physicochemical of ‘BRS Vitoria’ grape bunches and berries from different production cycles (PC2, PC3, and PC4). The column bars represent the mean ± standard deviation (*n* = 3). The different letters (a, b, c) indicate significant differences between production cycles (PC2, PC3, and PC4) according to ANOVA followed by the Tukey test (α = 0.05). GAE: gallic acid equivalent; mv-3,-5-diglc: malvidin-3,5-diglucoside.

The results of the basic physicochemical characterization are shown in Figure 1. There was a significant difference between the physicochemical parameters obtained for the ‘BRS Vitoria’ grapes of PC2, PC3, and PC4. The moisture content level was highest (*p* ≤ 0.05) for PC3 (80.76 ± 0.16%), followed by PC2 (80.02 ± 0.22%) and PC4 (79.13 ± 0.01%). These results are similar to those that Nishiyama-Hortense et al. [18] obtained for the same grape from southern Brazil (78.63%). The Aw, however, was significantly higher (*p* ≤ 0.05) for PC4 (0.985 ± 0.001) and PC3 (0.977 ± 0.001), although PC2 showed a similar value (0.974 ± 0.001).

The SS content varied from 17.33 ± 0.38 (PC3) to 18.92 ± 00.14 (PC4) ºBrix, being higher (*p* ≤ 0.05) for PC4 and PC2 (18.02 ± 0.02). The rootstock may have influenced these results, as both grape samples (from PC2 and PC4) were grown on the ‘Paulsen 1103’ rootstock. Calili et al. [10] may corroborate this finding, reporting that ‘BRS Vitoria’ grapes cultivated under subtropical conditions (São Palo, Brazil) on the ‘Paulsen 1103’ rootstock exhibited higher SS accumulation compared to those grown on the ‘IAC 572’ and ‘IAC 766’ rootstocks. However, Leão et al. [7] did not find a significant difference in the SS content for ‘BRS Vitoria’ grapes grown on the studied rootstocks in a semi-arid region of Brazil across different PCs, highlighting that multiple factors typically influence such variations.

Calili et al. [10] reported that regardless of the rootstock used, ‘BRS Vitoria’ grapes harvested during berry maturation during a year with higher precipitation exhibited lower SS levels, particularly when rainfall occurred closer to the harvest date. The authors noted that grape ripening typically occurs during the sunniest periods of the year, when higher temperatures favor sugar accumulation. Conversely, excessive rainfall during the ripening phase reduces the accumulation of soluble carbohydrates in the fruit. Furthermore, according to Yan et al. [22], the negative correlation between precipitation and SS may be explained by the fact that water stress diminishes the photosynthetic capacity of mature grape leaves. This stress alters the timing of dry matter accumulation during berry growth. It shifts the dry matter buildup period from the fruit-setting stage to the color-conversion stage, ultimately affecting sugar concentration.

Similarly, Anastasiou et al. [15] found a significant correlation between SS content and climatic parameters in a study on commercial table grapes from Greece. However, the strongest association was observed with growing degree days from flowering to bunch closure. In the present study, the combination of higher rainfall and lower average temperatures during PC3, compared to PC2 and PC4, may have contributed to the observed SS values. Independent of the PC, these results demonstrate that the studied ‘BRS Vitoria’ grapes could be marketed according to international trade standards, establishing that the minimum SS content for table grapes may vary from 14.0 to 17.5 ºBrix [8].

The TA values, expressed as tartaric acid g⋅100 g^−1^, were in decreasing order (*p* ≤ 0.05): 0.77 ± 0.01 (PC2) > 0.66 ± 0.01 (PC3) > 0.45 ± 0.01 (PC4), whereas pH values were inversely ordered: 4.06 ± 0.02 (PC4) > 3.91 ± 0.02 (PC3) > 3.85 ± 0.02 (PC2). Moreover, with these values, according to descriptor no. 506 [23], Table 3 shows that PC3 and PC4 were classified as low in acidity and PC2 as medium. Colombo et al. [3] reported a variation in the TA between 0.69 and 0.75 g tartaric acid⋅100 g^−1^ and pH between 4.62 and 4.69 for berries from bunches with different densities. It is plausible that climatic conditions have played a significant role in these results, as PC4 experienced the lowest rainfall and the highest average temperature. Contrary to the SS content in the present study, several authors have reported a strong positive correlation between precipitation and TA in grapes, with a corresponding negative correlation with pH [10,22] Hewitt et al. [24] observed that thermal stress during the maturation of two grape varieties (Cabernet Sauvignon and Riesling) led to a decrease in the TA, reducing the content of organic acids, especially when combined with water stress. This alteration is probably linked to the expression of genes that regulate the metabolism of these acids, as stress conditions can influence gene expression in metabolic pathways such as glycolysis and gluconeogenesis, thereby impacting the production and degradation of organic acids in the berries. In contrast, Anastasiou et al. [15], while evaluating the influence of soil conditions and climatic parameters on the quality of table grapes from Greece, found a strong correlation between only climatic parameters and pH, not with acidity. The authors emphasize that pH is influenced by multiple vineyard management practices, even though it is expected to increase under hot and dry conditions. These findings underscore that the fruit’s physicochemical parameters result from a multitude of factors, making their study complex and specific.

As a result of the significant differences found for the different PCs, the SS/TA also differed; the highest one (*p* ≤ 0.05) was found for PC4 (38.68 ± 0.58), followed by PC3 (26.62 ± 0.67) and PC2 (20.50 ± 0.10). As mentioned above (Item 2.1), all the grapes evaluated would be suitable for marketing since they had an SS/TA ratio greater than 20. However, it is interesting to note that the highest value was found for PC4 from the main harvest. Moreover, the reducing sugar (RS) content among the PCs varied between 13.64 ± 0.09 and 14.43 ± 0.11 g glucose⋅100 g^−1^, and was significantly higher for PC4 (*p* ≤ 0.05). No significant differences were found for the total sugar (TS) content (*p* > 0.05), which varied between 14.47 ± 0.14 and 14.75 ± 0.39 g glucose⋅100 g^−1^, classifying all PCs as low in sugar content (Table 3), according to descriptor no. 505 by OIV [23]. These results suggest that the lower acidity was mainly responsible for the highest SS/TA ratio obtained for PC4.

Finally, the total phenolic compound (TPC) and total monomeric anthocyanin (TMA) concentration in the berries from the different PCs varied between 1234.13 and 1750.88 mg GAE⋅kg^−1^ (*p* ≤ 0.05) and between 503.32 and 742.86 mg mv-3,5-diglc⋅kg^−1^ (*p* ≤ 0.05), respectively. Significant differences were found for both parameters, with the highest values found for grapes from PC4, followed by PC3 and PC2. In a recent study involving ‘BRS Vitoria’ grapes produced in Marialva, Paraná State, Brazil, Colombo et al. [3] reported a total anthocyanin content of approximately 597 mg⋅kg^−1^, within the range observed in the present study. In general, this concentration of anthocyanins in ‘BRS Vitoria’ gives the berry its attractive color. Notably, as well as the SS/TA ratio, the concentration of these compounds was higher for grapes from the main harvest (PC4).

Other studies have already reported the influence of rootstocks on the phenolic composition of grapes [25,26]. However, using different rootstocks in the present study did not appear to influence the results obtained, with the observed differences likely attributable to climatic conditions. In a study on Merlot grapes in Spain, Ramos et al. [27] reported that climatic variations affected different phenolic compounds in distinct ways. Specifically, the authors observed a decrease in the TMA concentration with increasing temperature, which contrasts with the present study’s findings, where the highest TMA content was detected in PC4, which had the highest average temperature. It is important to note that the influence of temperature on anthocyanin biosynthesis may depend on the phenological stage of grape development [27]. In the present study, the period of highest temperature within the berry growth phases was not controlled.

Additionally, among environmental factors, sunlight exposure is a critical stimulus regulating anthocyanin accumulation, with several studies reporting that anthocyanin content decreases when grapes are not exposed to direct sunlight [28,29,30]. Indeed, synthesizing secondary metabolites, particularly UV-absorbing flavonoids, is a protective response of grape berries to UV radiation stress [29]. Environmental factors can alter the expression of genes involved in anthocyanin biosynthesis and transport in a cultivar- and tissue-specific manner [28]. In this context, the results of the present study may be partially explained by the higher solar radiation incidence during the November PC, which occurs in the Brazilian summer, which presents longer sunlight exposure compared to the May PC (PC2 and PC3). This factor may have favored the biosynthesis of anthocyanins and other phenolic compounds, contributing to the higher levels of TPCs and TMAs observed in this period.

**Table 3:** Physicochemical and morphological characterization * of ‘BRS Vitoria’ grape bunches and berries from different production cycles (PC2, PC3, and PC4) according to pre-established descriptors and categories. Results are expressed numerically according to coded category or as mean ± standard deviation (classified category), as appropriate.

n°	Descriptor	Categories	PC2	PC3	PC4
502	Bunch mass (g) ^1^	1: Very low (≤100 g); 3: Low (≈300 g); 5: Medium (≈500 g); 7: High (≈700 g); 9: Very high (≥900).	221.40 b ± 37.94 (3)	295.05 a ± 22.55 (3)	206.27 b ± 32.92 (3)
202	Bunch length (mm) ^1^	1: Very short (≤80 mm); 3: Short (≈120 mm); 5: Medium (≈160 mm); 7: Long (≈200 mm); 9: Very long (≥240 mm).	134.34 b ± 14.49 (3)	161.93 a ± 15.17 (5)	154.68 a ± 10.81 (5)
203	Bunch width (mm) ^1^	1: Very narrow (≤40 mm); 3: Narrow (≈80 mm); 5: Medium (≈120 mm); 7: Wide (≈160 mm); 9: Very wide (≥200 mm).	68.37 a ± 6.73 (3)	67.25 a ± 4.93 (3)	57.13 b ± 4.59 (3)
-	Bunch compactness ^3^	1: Very loose; 3: Loose; 5: Full; 7: Moderately compact; 9: Very compact.	9	9	9
-	Bunch shape ^4^	1: Short tapered; 2: Tapered with shoulders; 3: Tapered; 4: Cylindrical; 5: Cylindrical winged; 6: Winged with double bunches.	4	4	4
227	Pruine ^1^	1: None or very low; 3: Low; 5: Medium; 7: High; 9: Very high.	7	7	9
503	Berry mass (g)^1^	1: Very low (up to about 1 g); 3: Low (about 3 g); 5: Medium (about 5 g); 7: High (about 7 g); 9: Very high (about 9 g or more).	3.77 b ± 0.28 (3)	4.57 a ± 0.29 (5)	2.86 c ± 0.20 (3)
-	Berry shape ^3^	1: Spherical; 2: Flat; 3: Ellipsoid; 4: Elongated; 5: Ovoid; 6: Oval; 7: Obovoid; 8: Elongated curved.	3	3	3
220	Berry length (mm) ^1^	1: Very short (up to about 8 mm); 3: Short (about 13 mm); 5: Medium (about 18 mm); 7: Long (about 23 mm); 9: Very long (about 28 mm or more).	21.42 b ± 0.63 (7)	22.80 a ± 0.53 (7)	19.22 c ± 1.39 (5)
221	Berry diameter (mm) ^1^	1: Very narrow (up to about 8 mm); 3: Narrow (about 13 mm); 5: Medium (about 18 mm); 7: Wide (about 23 mm); 9: Very wide (about 28 mm or more).	16.45 b ± 0.41 (5)	18.15 a ± 0.67 (5)	15.32 c ± 0.41 (3)
-	Berry diameter (mm) ^2^	10: <12 mm; 12: 12–14 mm; 14: 14–16 mm; 16: 16–18 mm; 18: 18–20 mm; 20: 20–22 mm; 22: 22–24 mm; 24: 24–26 mm; 26: 26–28 mm; 28: 28–30 mm; 30: 30–32 mm; 32: ≥32 mm.	16	18	14
222	Uniformity of berry size ^1^	1: Not uniform; 2: Uniform.	2	2	2
225	Skin color ^1^	1: Green-yellow; 2: Rose; 3: Red; 4: Grey; 5: Dark red violet; 6: Blue-black.	5	5	5
226	Uniformity of skin color ^1^	1: Not uniform; 2: Uniform.	2	2	2
231	Intensity of flesh coloration ^1^	1: None or very weak; 3: Weak; 5: Medium; 7: Strong; 9: Very strong.	1	1	1
241	Formation of seeds ^1^	1: None; 2: Rudimentary (incomplete embryo development); 3: Complete (perfectly developed seeds).	1	1	1
505	Sugar content ^1^	1: Very low (≤12%); 3: Low (≈15%); 5: Medium (≈18%); 7: High (≈21%); 9: Very high (≥24%).	14.59 a ± 0.27 (3)	14.47 a ± 0.14 (3)	14.75 a ± 0.39 (3)
506	Total acidity (g of tartaric acid⋅L^−1^) ^1^	1: Very low (3 g⋅L^−1^); 3: Low (≈6 g⋅L^−1^); 5: Medium (≈9 g⋅L^−1^); 7: High (≈15 g⋅L^−1^); 9: Very high (≥15 g⋅L^−1^).	7.69 a ± 0.05 (5)	6.55 b ± 0.01 (3)	4.51 c ± 0.81 (3)

Mean ± standard deviation * *n* = 12 for bunches, *n* = 120 for berries. Different letters in the same line (a, b, and c) indicate significant differences between production cycles (PC2, PC3, and PC4) according to ANOVA followed by the Tukey test (α = 0.05). ^1^ OIV [23]; ^2^ BRASIL [31]; ^3^ SOUSA [32]; and ^4^ WEAVER [33].

Table 3 shows the physicochemical and morphological characteristics of the bunches and berries of ‘BRS Vitoria’ grapes from PC2, PC3, and PC4, as well as the pre-established criteria and descriptors used. There was a significant difference among the PCs for the evaluated parameters. In all PCs, the cylindrical bunch shape was predominant, with bunch mass between 206.27 and 295.05 g, length between 134.34 and 161.93 mm, and width between 57.13 and 68.37 mm. With that, the bunches fall into the category of low mass, although the grapes from PC3 had a significantly higher mean mass value than PC2 and PC4; regarding length, PC3 and PC4 were characterized as medium and PC2 as short. For all PCs, the width was classified as narrow. These results corroborate others reported for this cultivar [6,8]. Leão et al. [6] investigated ‘BRS Vitoria’ in the Vale do São Francisco (State of Pernambuco, Brazil) and found a cylindrical shape, with mean values of 220 g for mass, 154 mm for length, and 75 mm for width. Bunch mass is an important quality attribute for the marketing of table grapes. Even though the bunches of the ‘BRS Vitoria’ grapes from the studied PCs were not as heavy and long as that of the ‘BRS Melodia’ grape—whose bunches had mass and length values of approximately 574 g and 204 mm, respectively—it has a bunch size and mass well-accepted by both the domestic and export markets [34].

Regarding bunch compactness, all PCs were classified as very compact. This result corroborates that already reported by Colombo et al. [3], who emphasize that despite its qualities, the ‘BRS Vitoria’ cultivar has the disadvantage of having very dense bunches, which can compromise the quality of the grapes. Thus, thinning techniques are necessary to control compactness, allowing berries to reach their maximum size. ‘BRS Vitoria’ grape berries may be classified as ideal for the table grape market when they are full and slightly loose. Based on visual and comparative observations among PCs, it was observed that all berries exhibited an ellipsoid shape. The bloom (pruine) present on the berry skin was visually detected in all PCs; PC2 and PC3 fell into category 7 (high), and PC4 into category 9 (very high) (descriptor no. 227, OIV [23], Table 3). The bloom is a natural wax that covers the grape berry, providing a physical barrier against pathogens and water loss [16]. Its absence can be classified as a mild defect, according to the Technical Regulation of Quality and Identity for table grape classification [31]. Nonetheless, if the grape is used for obtaining raisins, the pruine can delay the drying due to its hydrophobic nature, therefore impairing the sensory and nutritional quality of raisins, especially if high temperatures are used during the process [16].

The grape berries’ mass, length, and diameter varied from 2.86 (PC4) to 4.57 (PC3) g, 19.22 (PC4) to 22.80 (PC3) mm, and 15.32 (PC4) to 18.15 (PC3) mm, respectively. The mass values for grape berries from PC2 and PC4 fall into category 3 (low) and were significantly lower than PC3, which fall into category 5 (medium). Moreover, all assessed berries were classified as uniform (descriptor no. 222, OIV [23], Table 3). As for the size of the berries (length and diameter), those from PC2 and PC3 fall into related categories, classified as long (about 23 mm) and medium in diameter (about 18 mm). On the other hand, berries from PC4 were classified as “medium” in length (19 mm) and “narrow” in diameter (15 mm). Thus, the studied grapes from different PCs (PC2, PC3, and PC4) exhibited greater length than those previously reported by Maia et al. [8], who described the ‘BRS Vitoria’ grape as being 17 mm in length and 19 mm in diameter. Leão et al. [6] classified ‘BRS Vitoria’ grape berries with mean values of 3.70 g for mass and 22.49 and 16.78 mm, respectively, for length and diameter, which is very similar to those found in the present study. The berry diameter standard is another essential attribute for table grapes in international and domestic markets. In Brazil, the minimum required diameter is 12 mm; the ideal value, however, is between 14 and 17 mm for greater acceptance [31]. According to the Brazilian classification [31] for berry diameter, it can be assumed that berries from PC2, PC3, and PC4 were subclass 16, 18, and 14, respectively.

Overall, the grapes from PC3 stood out in terms of morphological characteristics, particularly regarding berry size and mass. This distinction may be attributed to the use of the ‘IAC 572’ rootstock, as it was the only cycle in this part of the study to utilize this particular rootstock. The root system plays a critical role in providing the plant with the necessary water and minerals for growth, as well as storing most of the nutrient reserves [35]. For the same cultivar, Leão et al. [7] reported a significant difference in berry mass across certain PCs, with berries from vines grown on the ‘Paulsen 1103’ rootstock being smaller compared to those grown on other rootstocks. However, it is important to note that climatic conditions may have also contributed to these results. It has been widely reported that weather conditions during the growing season greatly affect flowering, and consequently, the final quantitative characteristics of grape production. Anastasiou et al. [15] reported that rainfall has a positive effect on berry diameter, berry weight, and yield. Additionally, temperature seems to influence these parameters differently depending on the grapevine development stage. In the present study, PC3 exhibited one of the highest precipitation volumes during the PC, which may have contributed to the observed outcomes. However, regardless of the PC, the ‘BRS Vitoria’ grape characteristics were suitable for fresh fruit marketing and might also be appropriate for producing more oversized raisins marketed as healthy snacks.

Regarding the berry color, the grapes from different PCs were characterized as dark red, violet, and uniform (descriptors no. 225 and no. 226, OIV [23], Table 3). It should be noted that the grape’s attractive color and the skin color’s uniformity are crucial factors for consumer acceptance of fresh fruit and the commercial value of table grapes, such as derived products [34]. Some other characteristics observed in the cultivar from the different PCs assessed were thick and resistant skin with firm, colorless, seedless flesh; they have already been described by Leão et al. [6] and Maia et al. [8]. Therefore, for the intensity of flesh anthocyanin coloration and the formation of seeds, ‘BRS Vitoria’ grape berries fall into category 1: none or very weak and none, respectively (descriptors no. 231 and no. 241, OIV [23], Table 3). The absence of seeds is another factor that encourages the use of this cultivar as a raw material in producing unique products such as jams and raisins [16].

The percentages of berry skin and flesh from the grapes from different PCs were calculated and varied from 7.14% (PC4) to 9.49% (PC2) and from 90.51% (PC2) to 92.86% (PC4), respectively. This information may help when evaluating their quality for fresh consumption, along with other factors such as the skin’s crunchiness and the flesh’s juiciness. Other seedless grape cultivars have also had their skin and flesh ratio determined, such as ‘BRS Morena’ (25.0% skin and 75.0% flesh) and ‘BRS Clara’ (24.0% skin and 76.0% flesh) [36]. When comparing the results of these cultivars with the ones found in this study, one can observe that the ‘BRS Vitoria’ grape has a higher percentage of flesh than the skin, which increases fruit appreciation and adds value to its commercialization.

**Figure 2:**
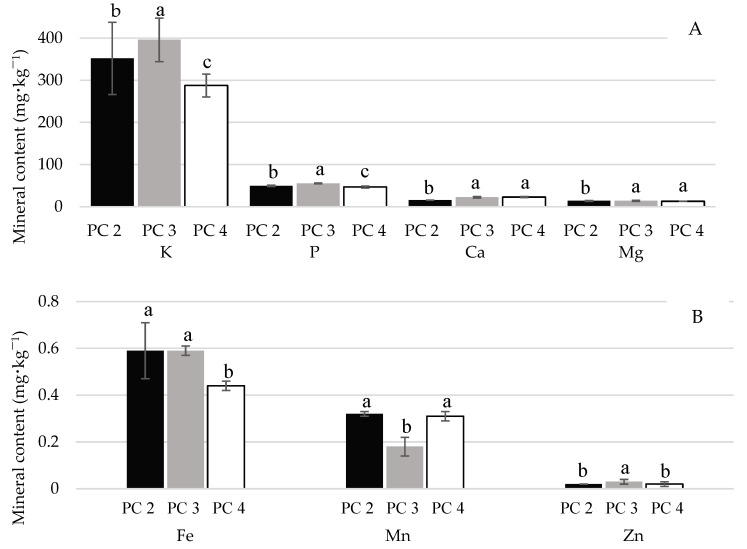
Mineral content of ‘BRS Vitoria’ grapes in different production cycles (PC2, PC3, and PC4). (**A**) Macrominerals: K: potassium, P: phosphorus, Ca: calcium, Mg: magnesium; and (**B**) Microminerals: Fe: iron, Mn: manganese, Zn: zinc. The column bars represent the mean ± standard deviation (*n* = 2). The different letters (a, b, c) indicate significant differences between production cycles (PC2, PC3, and PC4) according to ANOVA followed by the Tukey test (α = 0.05).

Figure 2 shows the macro- and micromineral contents found in the grapes from the different PCs PC2, PC3, and PC4. It is known that the mineral composition of grapes is influenced by external factors, such as soil composition, cultural practices, nutritional management strategies, and edaphoclimatic conditions during PCs [20]. Inherent characteristics, such as the cultivar, genotype, rootstock, age, depth of the root, and grape ripening, will also influence the vine. Significant differences (*p* ≤ 0.05) in the individual concentrations of some minerals could be observed. The concentration (mg⋅100 g^−1^) of macrominerals found in the studied grapes varied from 14 (Mg for PC2) to 396 (K for PC3), whereas that of microminerals varied from 0.02 (Zn for PC2 and PC4) to 0.59 (Fe for PC2 and PC3).

The concentration of K (288–398 mg⋅100 g^−1^) and P (47–55 mg⋅100 g^−1^) were the highest among the minerals analyzed. Both showed significant differences (*p* ≤ 0.05) across the PCs, with PC3 exhibiting the highest mean values and PC4 the lowest. This variation may be related to the use of these minerals to fertilize the soil where vines were grown and to the diversity of rootstocks used, as these may affect nutrient absorption by the vines [10,37]. According to Yan et al. [22], approximately 50% of K absorbed by the vine is accumulated in the grapes. For Ca (16–23 mg⋅100 g^−1^) and Mg (13–14 mg⋅100 g^−1^), the concentrations were significantly higher for PC3 and PC4, respectively. Based on these results, the PC with the highest concentration of macrominerals among the PCs evaluated in this study stage was PC3. Among the microminerals analyzed in the PCs for the ‘BRS Vitoria’ grape, the most abundant was Fe (0.44–0.55 mg⋅100 g^−1^), followed by Mn (0.18–0.32 mg⋅100 g^−1^). The lowest concentrations were observed for Zn (0.02–0.03 mg⋅100 g^−1^).

The higher values of minerals, particularly macronutrients, found in the grapes from PC3 may indicate that the ‘IAC 572’ rootstock promoted greater nutrient content and nutrient accumulation in the vines, allowing for increased transfer to the fruit. These findings are consistent with those reported by Callili et al. [38], who, for the same cultivar, observed that grapevines grafted onto ‘IAC 572’ and ‘IAC 766’ exhibited higher concentrations of N, K, and Ca than those grafted onto ‘Paulsen 1103’. The authors highlighted that the increased productivity observed in vines grafted onto ‘IAC 572’ may be intrinsically linked to the higher nutrient content in the bunches or shoots. The choice of rootstock influenced the amount of nutrients removed from the vineyard through pruning and bunch harvest, with vines grafted onto ‘IAC 572’ demonstrating higher nutrient export rates than those grafted onto ‘Paulsen 1103’. Regardless of rootstock, the nutrient export order remained consistent: K > N > Ca > P > Mg > S > Mn > Fe > B > Zn > Cu. These results corroborate those found in the present study, reinforcing the possibility of ‘IAC 572’ enhancing nutrient uptake and translocation to the grapevines. However, it is important to note that the rootstock alone is not the sole factor influencing nutrient content, as environmental conditions may have also played a role in the observed results.

**Figure 3:**
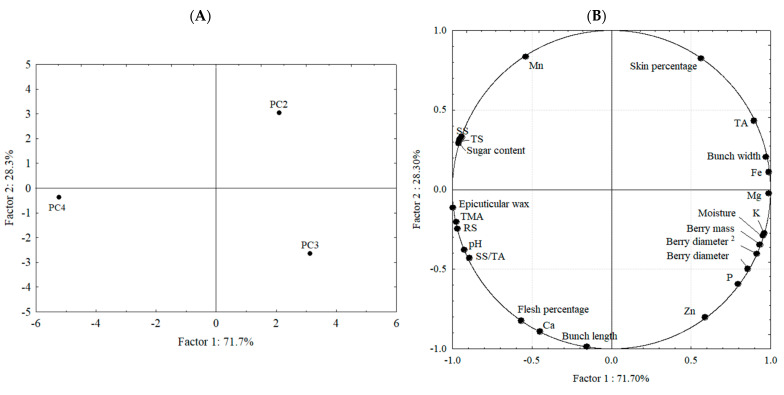
The principal component analysis for the physicochemical, morphological, and mineral characterization of the ‘BRS Vitoria’ grapes from different production cycles (PC2, PC3, and PC4). (**A**) Projection of the studied cycles; and (**B**) projection of variables. ^2^: BRASIL [31], TMA: Total monomeric anthocyanin, TA: total acidity, TS: total sugar, RS: reducing sugar, SS: soluble solids, K: potassium, P: phosphorus, Mg: magnesium, Fe: iron, Mn: manganese, Ca: calcium, and Zn: zinc.

To evaluate any possible correlation between all the parameters studied for the ‘BRS Vitoria’ grapes from PC2, PC3, and PC4, a principal component analysis (PCA) was carried out (Figure 3). The first (Factor 1) and second (Factor 2) principal components explained, respectively, 71.7 and 28.3% of the data variation.

The PCA shows a clear separation between the samples, corroborating what was previously presented and discussed in this paper. It can be seen that the grapes from PC4 were mainly described by the chemical parameters related to the sugar content of the sample (SS, RS, and TS), as well as pH, the prominent presence of epicuticular wax and, in particular, the SS/TA ratio and the amount of TMAs. Therefore, it should be noted that the grapes from the main harvest (PC4) stood out in terms of parameters that are important for the taste and nutritional quality of the fruit. The development of these characteristics may be primarily attributed to the environmental conditions during the period, including lower rainfall, higher average temperature, and increased sunlight exposure, typical of the summer season. However, they were negatively correlated with parameters important for the appearance, especially related to its size and mass, and with mineral content (K, P, Mg, and Fe). It is noteworthy that Ramos et al. [27], in a study on Merlot grapes in Spain, reported a positive correlation between berry mass and TA and a negative correlation between berry mass and sugar content, findings that align with those of the present study. The authors further observed that both berry mass and TA decreased with increasing maximum temperature—both during the flowering-to-veraison period and from veraison to maturation—while sugar content increased. The results for PC4 corroborate those reported by Ramos et al. [27], as this cycle exhibited the highest maximum and average temperatures.

These parameters (related to size, mass, and mineral content) were the main descriptors of the PC3 grapes, which originated from the ‘IAC 572’ rootstocks. As previously mentioned, the lower values of sugar and pH in the grapes from this rootstock may have been influenced by either the rootstock itself or environmental conditions, such as higher precipitation and lower average temperatures. On the other hand, the higher mineral content could have been a result of the influence of the rootstock, as previously reported for grapes of the same cultivar [38]. It is also worth noting the positive correlation between these parameters. Calili et al. [38] emphasize that in viticulture, each nutrient has a specific function, with potassium, for example, being essential for characteristics such as yield and berry size. The grapes from PC2 (from ‘Paulsen 1103’ rootstocks and second harvest), were primarily characterized by their skin percentage and TA.

Yan et al. [22] found a significant correlation between soil K content and *Vitis vinifera* grapes’ TA in China. They suggested this could be due to the higher K+ content in grape berries, which influences the acidity levels in vacuoles. In the present study, a positive correlation between K content and TA was also observed in the PCA analysis. However, due to this study’s limitations, it is not possible to determine whether higher mineral content in the soil or climatic factors primarily influenced this correlation. Notably, PC4, which produced grapes with lower TA and mineral content, such as K, had the lowest precipitation and the highest average temperature throughout the entire production cycle.

As discussed throughout the article, the differences observed between the PCs are likely attributed to the distinct rootstocks used and the variations in edaphoclimatic conditions across the harvests, with each characteristic—physicochemical, morphological, phenolic, and mineral composition—being distinctly affected. It should also be noted that, as mentioned earlier and elaborated throughout the article, the grapes’ physicochemical, morphological, and nutritional characteristics depend on various factors, most likely involving a synergistic effect between them [24].

## 3. Materials and Methods

### 3.1. Reagents and Standards

All solutions were made of deionized water (18.2 MΩ cm, Milli-Q, Millipore, Bedford, MA, USA). All solvents were HPLC-grade, and all chemicals were analytical grade (99%). The standards used for anthocyanin analyses were mv-3,5-diglc, cy-3,5-diglc, pn-3-glc, cy-3-glc, purchased from Sigma-Aldrich (Barcelona, Spain), mv-3-glc (Extrasynthese, Genay, France), and pn-3,5-diglc (Phytolab, Vestenbergsgreuth, Germany). For mineral analysis, nitric acid (65%, *w*/*w*) and hydrogen peroxide (30%, *w*/*w*), both from Merck (Darmstadt, Germany), were used as digestion solvents. The calibration curves were constructed using a multi-element standard solution (10 mg⋅mL^−1^, Perkin Elmer, Waltham, MA, USA).

### 3.2. Grape Samples

**Figure 4:**
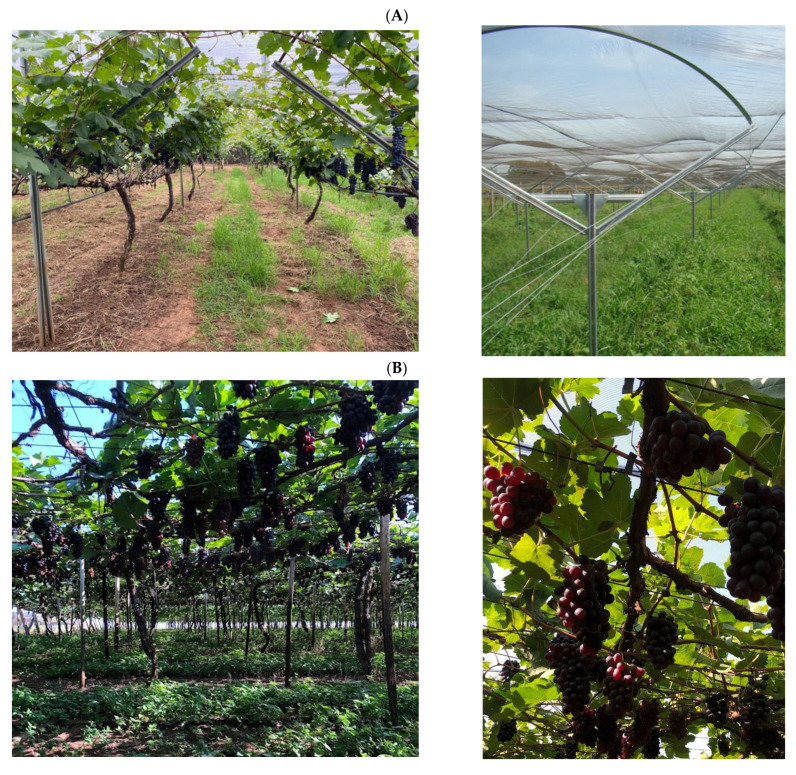
Planting systems of ‘BRS Vitoria’ grape in northwest São Paulo, Brazil. (**A**): ‘Y’-shaped system and (**B**): trellis system.

Representative batches (≈30 kg) from four consecutive PCs of ‘BRS Vitoria’ grapes (PC1, PC2, PC3, and PC4) were collected from the northwestern region of São Paulo State, Brazil (20°09′27″ S, 50°37′55″ W). The climate is tropical with a dry winter, and the soil is classified as red-yellow clay soil (Ultisol equivalent) according to the USDA Soil Taxonomy [39]. The bunches were sampled in four different harvests following a zigzag path between two marked rows of vines in three different zones of the vineyard. The ‘BRS Vitoria’ grapes were grafted onto two rootstocks: ‘IAC 572’, trained on a trellis system, with average yields of 30–32 tons per hectare, and ‘Paulsen 1103’, trained on a ‘Y’-shaped system, with average yields of 23.9–25 tons per hectare. The trellis and ‘Y’-shaped system are shown in the Figure 4. Due to the experimental stage of viticulture for this cultivar in the region and limitations in available material, it was not possible to obtain all four harvests with both rootstocks. Consequently, grapes grafted onto ‘IAC 572’ were analyzed during the first (PC1, main harvest, harvested in November 2018) and third (PC3, second harvest, harvested in May 2020) production cycles, while those grafted onto ‘Paulsen 1103’ were examined during the second (PC2, second harvest, harvested in May 2019) and fourth (PC4, main harvest, harvested in November 2020) cycles. This alternation was adopted to ensure that both rootstocks were evaluated throughout the study period, providing an initial understanding of their influence on grape composition.

**Figure 5:**
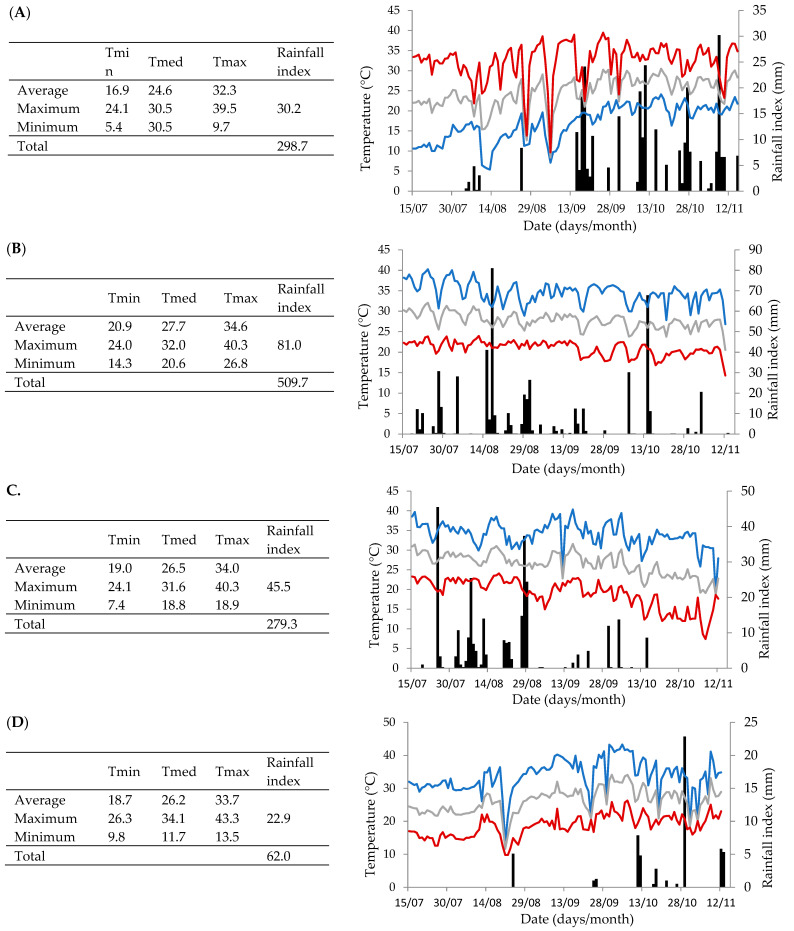
The recordings of meteorological data during the cultivation of ‘BRS Vitoria’ grapes in four production cycles (PC1, PC2, PC3, and PC4). The line graph represents the following: Tmin (red): minimum temperature (°C); Tmed (grey): average temperature (°C); and Tmax (blue): maximum temperature (°C). The bar chart represents the rainfall index (mm). (**A**): First production cycle (PC1), harvested November 2018; (**B**): Second production cycle (PC2), harvested May 2019; (**C**): Third production cycle (PC3), harvested May 2020; (**D**): Fourth production cycle (PC4), harvest November 2020.

The northwest region of São Paulo has a tropical climate, providing sufficient thermal availability for year-round grape production. Irrigation was performed using a micro-sprinkler system. To protect against bird and bat damage, the plants were covered with black polyethylene netting providing 18% shading. Fungal disease control was carried out preventively following a fixed calendar, as commonly practiced in the region, through applications of protective and systemic fungicides registered for grapevines. These applications were adjusted based on meteorological conditions and the phenological stage of the plants (Figure 5), according to CIIAGRO [40].

### 3.3. Physicochemical Characterization and Profiling of Minerals and Anthocyanins of ‘BRS Vitoria’ Grape

For ‘BRS Vitoria’ grapes from PC1, physicochemical and morphological characteristics were determined in triplicate according to the Association of Official Analytical Chemists [41]. Representative berries from the grape bunches were selected, destemmed, and homogenized with a mixer (PMX 600, Philco, São Paulo, Brazil). Moisture content was determined gravimetrically with a vacuum oven at 70 °C (Marconi, Piracicaba, Brazil); SS were measured using an ABBE refractometer (RTA-100, Quimib, São Paulo, Brazil), with results expressed in ºBrix (25 °C); pH and TA were assessed with a pH meter (Tec-5, Tecnal, Piracicaba, Brazil), with TA expressed as g tartaric acid100 g^−1^. The maturity index was calculated as the ratio of SS to TA.

A detailed qualitative and quantitative analysis of anthocyanins and mineral composition was performed to evaluate the potential of ‘BRS Vitoria’ grapes as raw material for derived products. Anthocyanin extraction followed the method described by Lago-Vanzela et al. [36], and separation, identification, and quantification were conducted using previously described methods [42]. The extract was filtered through a Chromafil PET 20/25 polyester membrane (0.20 μm, Macherey-Nagel, Düren, Germany) and injected (10 μL) directly into a Zorbax Eclipse XDB-C18 reversed-phase column (150 mm × 2.1 mm; 3.5 μm particle size, Agilent Technologies, Santa Clara, CA, USA), maintained at 40 °C. The Agilent 1100 Series system HPLC (Agilent Technologies, Santa Clara, USA, and Macherey-Nagel, Düren, Germany)—equipped with a diode array detector (DAD; G1315B) and an LC/MSD Trap VL (G2445C VL) electrospray ionization mass spectrometry (ESI-MS/MS) system—was used in positive ionization coupled to an Agilent ChemStation (version B.01.03) data-processing unit. The mass spectra data were analyzed using the Agilent LC/MS Trap software (version 5.3).

The identification was primarily based on spectroscopic data (UV–vis and MS/MS) for authentic standards (detailed in Section 3.1) or data from previous reports [42,43]. Quantification was performed using DAD-chromatograms extracted at 520 nm. Anthocyanin content was expressed as molar ratios (%) normalized to the total content, and the total anthocyanin concentration was reported as mg equivalents of mv-3-glc and mv-3,5-glc⋅kg^−1^ of fresh weight (FW) grapes. For this purpose, external calibration curves were employed, covering the expected concentration ranges of 7.62–374.46 mg mv-3-glc⋅L^−1^ and 4.94–239.17 mg mv-3,5-glc⋅L^−1^.

For the mineral analysis, K, P, Ca, Mg, Fe, Mg, and Zn were quantified following the AOAC method 2015.06 described by Pacquette et al. [44]. Homogenized grape samples (0.25 g, in duplicate) were transferred to poly-tetrafluoroethylene-co-perfluoropropyl vinyl ether–PFA (Teflon™) tubes and digested with a solution of 65% nitric acid and 30% hydrogen peroxide in a closed microwave system (Multiwave GO, Anton Paar, Torrance, USA) at 190 °C. After cooling, the extracts were filtered into 50 mL glass with their volume completed with ultrapure standard water (Milli-Q element, 18 MΩ cm^−1^). Internal standards (scandium for K, Ca, Fe, and Mg; germanium for P, Mn, and Zn; from Perkin Elmer, Waltham, MA, USA) were added for ICP-MS (NexION 1000 ICP-MS, Perkin Elmer, Waltham, MA, USA) analysis. The standard mode was used for K, Ca, and Mg, and the kinetic energy discrimination for the other minerals, with helium as the collision gas. The isotopes analyzed were 39K, 31P, 43Ca, 24Mg, 57Fe, 55Mn, and 66Zn. Calibration curves were prepared using standard stock solutions, and results were expressed in mg⋅100 g^−1^ of grapes.

### 3.4. Determination of Physicochemical and Morphological Characteristics and Mineral Compounds of ‘BRS Vitoria’ Grapes in Three PCs

In this second stage of the study, three PCs (PC2, PC3, and PC4) were evaluated for some physicochemical and morphological characteristics and mineral composition, determined by ICP-MS, as described in item 2.3. Therefore, twelve representative bunches from each PC were collected. Posteriorly, ten representative berries from each of the bunches were selected and classified according to nineteen descriptors pre-established by the Ministry of Agriculture, Livestock and Food Supply (MAPA) [31], Organisation Internationale de la Vigne et du Vin [23], Sousa [32], and Weaver [33]. The morphological analyses of bunches and berries were carried out by measuring and assessing (a total of one hundred and twenty berries per PC) with the aid of a digital analytical balance (AY220, Shimadzu, Barueri, Brazil) and a digital caliper (King Tools, Shanghai, China). The mean percentages (%, *w*/*w*) of the skin and flesh of the berries were determined by separating them with a stainless-steel knife and weighing them in the analytical balance (AY220, Shimadzu, Baureri, Brazil). The corresponding percentages were calculated based on the total mass of each berry analyzed.

Berries from these bunches were posteriorly crushed and homogenized for the chemical analyses in triplicate, according to methodologies recommended by the AOAC [41]: moisture content, SS, pH, TA, and SS/TA, as previously described; Aw, determined in an Aqualab analyzer at 25 °C (Addium Meter Group, São José dos Campos, Brazil); and RS and TS, with the results expressed in g glucose⋅100 g^−1^ of grape. Finally, with a spectrophotometer Model Agilent Technologies Cary 60 UV-Vis (Santa Clara, CA, USA), the TPCs were determined by the Folin–Ciocalteu method [45], with results expressed in mg equivalent gallic acid (EGA)⋅kg^−1^ of grape, and TMAs were determined by the method described by Ribereau-Gayon and Stonestreet [46], with results expressed in mg mv-3,5-glc⋅kg^−1^. For the TPCs and TMAs, it was necessary to conduct prior extraction of the compounds of interest, according to the methodology described by Lago-Vanzela et al. [36].

### 3.5. Statistical Analysis

All results were expressed as the mean ± standard deviation. The results were subject to an analysis of variance (ANOVA) followed by the Tukey test (α = 0.05) to compare the results from different PCs (PC2, PC3, and PC4) (the ANOVA table is provided in the Supplementary File (Table S1)), and a principal component analysis (PCA) was carried out. For the PCA, only the parameters that correlated strongly (factor loading > 0.8 or <−0.8) with the principal components were represented to better visualize the results. The software used for the PCA and all statistical analyses was Statistica^®^ 10.0 (Statsoft, Tulsa, OK, USA).

## 4. Conclusions

The results of this study provide initial insights into the influence of different rootstocks on the physicochemical, morphological, phenolic content, and mineral composition of ‘BRS Vitoria’ grapes across multiple PCs. Berries from PC1 presented an optimal ripening stage, specific mineral content, and a diverse anthocyanin profile, with mv being the predominant anthocyanidin. Grapes from the other production cycles (PC2, PC3, and PC4) also exhibited desirable characteristics for fresh consumption and potential as a nutraceutical ingredient. Environmental factors appeared to have a stronger impact on physicochemical traits, including the content of total phenolic compounds and anthocyanins, while the rootstock influenced mineral content and morphological characteristics. The study of the influence of these factors is complex due to the impact of various other variables and the synergistic effect between them. However, a direct comparison between rootstocks in all harvests was not feasible due to experimental limitations. Future studies should evaluate both rootstocks simultaneously and over more harvests to better understand their long-term effects. Investigating additional factors like soil and climate variability could further clarify the interactions between rootstock, PC, and grape quality, helping producers optimize growing and marketing strategies.

## Figures and Tables

**Table 1:** The ‘BRS Vitoria’ grape’s mineral composition * (mean ± standard) from the first production cycle (PC1).

Minerals	Concentration (mg⋅100 g^−1^)	Minerals	Concentration (mg⋅100 g^−1^)
Macrominerals		Microminerals	
K	345.16 ± 104.89	Fe	0.54 ± 0.14
P	50.50 ± 4.76	Mn	0.27 ± 0.08
Ca	20.34 ± 4.10	Zn	0.03 ± 0.01
Mg	13.61 ± 1.41		

* *n* = 3. K: potassium, P: phosphorus, Ca: calcium, Mg: magnesium, Fe: iron, Mn: manganese, and Zn: zinc.

**Table 2:** Anthocyanins (mean ± standard) by HPLC–DAD–ESI-MS/MS (positive ionization mode) for the ‘BRS Vitoria’ grape from the first production cycle (PC1): mass spectrum data, molar ratio, and total anthocyanin concentration (as equivalents of mv-3-glc and mv-3,5-diglc).

Anthocyanin *	Molecular Ion; Product Ion (*m*/*z*)	Molar Ratio (%)
dp-3,5-diglc	627; 465, 303	0.90 ± 0.05
cy-3,5-diglc	611; 449, 287	0.41 ± 0.04
pt-3,5-diglc	641; 479; 317	1.68 ± 0.15
pn-3,5-diglc	625; 463; 301	6.74 ± 0.52
mv-3,5-diglc	655; 493; 331	16.30 ± 2.06
dp-3-cmglc-5-glc	773; 611, 465, 303	2.43 ± 0.21
cy-3-cmglc-5-glc	757; 595, 449, 287	0.33 ± 0.21
pt-3-cmglc-5-glc	787; 625;317	3.35 ± 1.29
pn-3-cmglc-5-glc	771; 609; 307	2.81 ± 0.72
mv-3-*trans-*cmglc-5-glc	801; 639; 331	16.94 ± 2.71
mv-3-*cis-*cmglc-5-glc	801; 639; 331	1.59 ± 1.06
mv-3-acglc-5-glc	697; 535; 493; 331	1.06 ± 0.33
dp-3-*trans*-glc	465; 303	0.21 ± 0.05
dp-3-*cis*-glc	465; 303	4.47 ± 1.36
cy-3-glc	449; 287	1.97 ± 0.42
pt-3-*trans*-glc	479; 317	0.79 ± 0.16
pt-3-*cis*-glc	479; 317	3.51 ± 0.87
pn-3-glc	463; 301	2.60 ± 0.54
mv-3-glc	493; 331	5.10 ± 1.17
dp-3-acglc	507; 303	0.30 ± 0.13
cy-3-acglc	491; 287	0.48 ± 0.20
pt-3-acglc	521; 317	0.30 ± 0.10
pn-3-acglc	505; 301	0.29 ± 0.12
mv-3-acglc	535; 331	1.64 ± 0.17
dp-3-*trans*-cmglc	611; 303	6.47 ± 1.02
cy-3-*trans-*cmglc	595; 287	2.65 ± 0.30
pt-3-*trans-*cmglc	625; 317	4.58 ± 0.59
pt-3-*cis*-cmglc	625; 317	0.17 ± 0.01
pn-3-*trans*-cmglc	609; 301	2.11 ± 0.24
pn-3-*cis*-cmglc	609; 301	0.12 ± 0.03
mv-3-*trans*-cmglc	639; 331	7.24 ± 0.88
mv-3-*cis*-cmglc	639; 331	0.22 ± 0.01
pl-3-cmglc	433; 271	0.02 ± 0.00
Total Anthocyanin Concentration (mv-3-glc) (mg⋅kg^−1^)		135.59 ±14.96
Total Anthocyanin Concentration (mv-3,5-diglc) (mg⋅kg^−1^)		202.28 ± 22.31

* dp: delphinidin, cy: cyanidin, mv: malvidin, glc: glucoside, acglc: 6″-(acetyl)glucoside, cmglc: 6″-(p-coumaroyl) glucoside, pn: peonidin, pt: petunidin, *trans: trans* configuration, and *cis*: *cis* configuration. *n* = 3.

## Data Availability

The original contributions presented in this study are included in the article. Further inquiries can be directed to the corresponding author.

## Supplementary Materials

The following supporting information can be downloaded at: https://www.mdpi.com/article/10.3390/plants14060949/s1. Table S1. ANOVA tables for the physicochemical and morphological characterization of ‘BRS Vitoria’ grapes in different production cycles. SS: Sum of squares, MS: Mean Square, *F*: *F*-statistic.

## Abbreviations

PC	Production cycle
SS	Soluble solids
TA	Total acidity
Aw	Water activity
TS	Total sugar
RS	Reducing sugar
TMA	Total monomeric anthocyanin
TPC	Total phenolic compound
mv	Malvidin
pn	Peonidin
pt	Petunidin
dp	Delphinidin
cy	Cyanidin
glc	Glucoside
K	Potassium
P	Phosphorus
Ca	Calcium
Mg	Magnesium
Mn	Manganese
Zn	Zinc
Fe	Iron
