# Supplementary material for: ‘BRS Vitoria’ Grapes Across Four Production Cycles: Morphological, Mineral, and Phenolic Changes
Source: Plants (Basel). 2025 Mar 18;14(6):949. doi: 10.3390/plants14060949 (PMC11944994; doi:10.3390/plants14060949)
Supplement: Supplementary file 1 [file plants-14-00949-s001.zip › plants-3509195-supplementary.pdf]

Table S1. ANOVA tables for the physicochemical and morphological characterization of 'BRS Vitoria' grapes in different production cycles. SS: Sum of squares, MS: Mean Square, *F*: *F*-statistic.

a. ANOVA table of the characterization of moisture of berries and bunches of ‘BRS Vitoria’ grapes from different production cycles (PC2, PC3 and PC4).

	**SS**	**Degrees of freedom**	**MS**	***F***	***p*-value**
**Intercept**	57557.08	1	57557.08	2268202	0.000000
**CP**	4.00	2	2.00	79	0.000049
**Erro**	0.15	6	0.03		
**Total**	4.15	8			

b. ANOVA table of the characterization of soluble solids (SS) of berries and bunches of ‘BRS Vitoria’ grapes from different production cycles (PC2, PC3 and PC4).

	**SS**	**Degrees of freedom**	**MS**	***F***	***p*-value**
**Intercept**	2945.233	1	2945.233	52887.26	0.000000
**CP**	3.782	2	1.891	33.96	0.000535
**Erro**	0.334	6	0.056		
**Total**	4.117	8			

c. ANOVA table of the characterization of total acidity (TA) of berries and bunches of ‘BRS Vitoria’ grapes from different production cycles (PC2, PC3 and PC4).

	**SS**	**Degrees of freedom**	**MS**	***F***	***p*-value**
**Intercept**	3.517971	1	3.517971	161620.4	0.000000
**CP**	0.155787	2	0.077893	3578.5	0.000000
**Erro**	0.000131	6	0.000022		
**Total**	0.155918	8			

d. ANOVA table of the characterization of pH of berries and bunches of ‘BRS Vitoria’ grapes from different production cycles (PC2, PC3 and PC4).

	**SS**	**Degrees of freedom**	**MS**	***F***	***p*-value**
**Intercept**	139.9489	1	139.9489	466496.3	0.000000
**CP**	0.0702	2	0.0351	117.0	0.000016
**Erro**	0.0018	6	0.0003		
**Total**	0.072	8			

e. ANOVA table of the characterization of total phenolic compounds (TPC) of berries and bunches of 'BRS Vitoria' grapes from different production cycles (PC2, PC3 and PC4).

	SS	Degrees of freedom	MS	*F*	*p*-value
**Intercept**	36515194	1	36515194	106807.0	0.000000
**CP**	968565	2	484283	1416.5	0.000000
**Erro**	5128	15	342		
**Total**	973693	18			

f. ANOVA table of the characterization of total monomeric anthocyanins (TMA) of berries and bunches of 'BRS Vitoria' grapes from different production cycles (PC2, PC3 and PC4).

	SS	Degrees of freedom	MS	*F*	*p*-value
**Intercept**	9593091	1	9593091	28163.43	0.000000
**CP**	233534	2	116767	342.81	0.000000
**Erro**	7153	21	341		
**Total**	240687	24			

g. ANOVA table of morphological characterization of the of bunch mass (g) of 'BRS Vitoria' grapes from different production cycles (PC2, PC3 and PC4) according to pre-established descriptors and categories.

	SS	Degrees of freedom	MS	*F*	*p*-value
**Intercept**	2089292	1	2089292	2067.059	0.000000
**CP**	54145	2	27072	26.784	0.000000
**Erro**	33355	33	1011		
**Total**	87500	35			

h. ANOVA table of morphological characterization of the of bunches lenght (mm) of 'BRS Vitoria' grapes from different production cycles (PC2, PC3 and PC4) according to pre-established descriptors and categories.

	SS	Degrees of freedom	MS	*F*	*p*-value
**Intercept**	813405.6	1	813405.6	4380.965	0.000000
**CP**	4907.1	2	2453.5	13.215	0.000061
**Erro**	6127.0	33	185.7		
**Total**	11034.1	35			

i. ANOVA table of morphological characterization of the of bunches width (mm) of 'BRS Vitoria' grapes from different production cycles (PC2, PC3 and PC4) according to pre-established descriptors and categories.

	SS	Degrees of freedom	MS	*F*	*p*-value
**Intercept**	148600.0	1	148600.0	4916.924	0.000000
**CP**	919.7	2	459.8	15.215	0.000021
**Erro**	997.3	33			
**Total**	1917	35			

j. ANOVA table of morphological characterization of the of berry mass (g) of 'BRS Vitoria' grapes from different production cycles (PC2, PC3 and PC4) according to pre-established descriptors and categories.

	SS	Degrees of freedom	MS	*F*	*p*-value
**Intercept**	501.4795	1	501.4795	7452.796	0.000000
**CP**	17.4398	2	8.7199	129.592	0.000000
**Erro**	2.2205	33	0.0673		
**Total**	19.6603	35			

k. ANOVA table of morphological characterization of the of berries lenght (mm) of 'BRS Vitoria' grapes from different production cycles (PC2, PC3 and PC4) according to pre-established descriptors and categories.

	SS	Degrees of freedom	MS	*F*	*p*-value
**Intercept**	16098.93	1	16098.93	18542.22	0.000000
**CP**	78.04	2	39.02	44.94	0.000000
**Erro**	28.65	33	0.87		
**Total**	106.69	35			

l. ANOVA table of morphological characterization of the of berries diameter (mm) of 'BRS Vitoria' grapes from different production cycles (PC2, PC3 and PC4) according to pre-established descriptors and categories.

	SS	Degrees of freedom	MS	*F*	*p*-value
**Intercept**	9965.330	1	9965.330	37973.96	0.000000
**CP**	48.693	2	24.347	92.78	0.000000
**Erro**	8.660	33	0.262		
**Total**	57.353	35			

m. ANOVA table of morphological characterization of the of berries diameter (mm) of 'BRS Vitoria' grapes from different production cycles (PC2, PC3 and PC4) according to pre-established descriptors and categories.

	SS	Degrees of freedom	MS	*F*	*p*-value
**Intercept**	9965.330	1	9965.330	37973.96	0.000000
**CP**	48.693	2	24.347	92.78	0.000000
**Erro**	8.660	33	0.262		
**Total**	57.353	35			

n. ANOVA table of the characterization of total sugar (TS) of berries and bunches of 'BRS Vitoria' grapes from different production cycles (PC2, PC3 and PC4).

	SS	Degrees of freedom	MS	*F*	*p*-value
**Intercept**	1749.313	1	1749.313	177892.3	0.000000
**CP**	1.072	2	0.536	54.5	0.000142
**Erro**	0.059	6	0.010		
**Total**	1072.06	8			

o. ANOVA table of the characterization of reducing sugar (RS) of berries and bunches of 'BRS Vitoria' grapes from different production cycles (PC2, PC3 and PC4).

	SS	Degrees of freedom	MS	*F*	*p*-value
**Intercept**	1918.947	1	1918.947	23231.32	0.000000
**CP**	0.119	2	0.060	0.72	0.523901
**Erro**	0.496	6	0.083		
**Total**	0.615	8			
